# Hemoptysis requiring bronchial artery embolization in patients with nontuberculous mycobacterial lung disease

**DOI:** 10.1186/s12890-019-0881-z

**Published:** 2019-06-27

**Authors:** Su Hwan Lee, Jin Hwa Lee, Jung Hyun Chang, Soo Jung Kim, Hee-Young Yoon, Sung Shine Shim, Min Uk Kim, Sun Young Choi, Yon Ju Ryu

**Affiliations:** 1 Division of Pulmonology, Department of Internal Medicine, Severance Hospital, Yonsei University College of Medicine, Seoul, Republic of Korea; 2 Division of Pulmonary and Critical Care Medicine, Department of Internal Medicine, College of Medicine, Ewha Womans University, 1071 Anyangcheon-ro, Yangcheon-gu, Seoul, 07985 Republic of Korea; 3 Department of Radiology, College of Medicine, Ewha Womans University, Seoul, Republic of Korea; 4 Department of Radiology, Seoul National University Boramae Medical Center, Seoul, Republic of Korea

**Keywords:** Mycobacterium infections, nontuberculous, Tuberculosis, Hemoptysis, Embolization, therapeutic

## Abstract

**Background:**

Although infections caused by nontuberculous mycobacteria (NTM) are increasing in prevalence, there are few data about hemoptysis in patients with NTM lung disease. This study investigated the characteristics and prognosis of hemoptysis secondary to NTM infection.

**Methods:**

Following a retrospective review of cases managed between 2006 and 2016, 183 patients with NTM lung disease were enrolled and analyzed.

**Results:**

Among 183 patients with NTM lung disease, *Mycobacterium intracellulare* (*n* = 64, 35%) was the major cause of NTM infection, followed by *M. avium* (*n* = 59, 32.2%) and *M. abscessus* complex (*n* = 40, 21.9%). Hemoptysis developed in 78 patients (42.6%), among whom 33 (42.3%) required bronchial artery embolization (BAE). Between patients with and without hemoptysis, there were no significant differences with respect to sex, radiographic manifestations, distribution over 3 lobes on chest computed tomography, history of pulmonary tuberculosis, antiplatelet or anticoagulation therapy, and species of NTM. However, mean age at diagnosis was significantly lower in the hemoptysis group in univariate and multivariate analyses (65.7 ± 12.8 vs. 59.7 ± 11.8, *P* = 0.002, odds ratio: 0.969, 95% confidence interval: 0.944–0.996). Among patients with hemoptysis, those requiring medical therapy and those requiring BAE were not significantly different in terms of demographic characteristics, radiographic manifestations, and distribution over 3 lobes. All patients who received BAE showed immediate clinical improvement, no procedure-related complications, and none of them died during the period under review.

**Conclusions:**

NTM lung disease patients commonly experienced hemoptysis without specific risk factors except for relatively young age. Although some patients with hemoptysis needed BAE, the success rate of BAE was high, and there were no serious complications associated with BAE.

## Background

The prevalence of human infection with nontuberculous mycobacteria (NTM), an emerging cause of chronic pulmonary disease, is gradually increasing worldwide [1, 2]. Most NTM have low pathogenicity, and they are widely distributed in natural environments, such as soil and water sources [3]. It is difficult to distinguish among NTM colonization, contamination, and infection. The American Thoracic Society (ATS) and the Infectious Disease Society of America (IDSA) reported diagnosis criteria for NTM infection with composite and complex contents in 2007 [4].

There are more than 150 known NTM species, and new species have recently been discovered [3]. Although there are regional differences, *Mycobacterium avium* complex, *Mycobacterium kansasii, and Mycobacterium abscessus* complex (MABSC) are known major pathogens associated with pulmonary diseases [5, 6]. The initiation of treatment for NTM disease depends on various factors, such as individual clinical course, comorbidities, and adverse effects of antimycobacterial therapy. Furthermore, long-term treatment is required after initiation [4]. Hemoptysis is among the various potential clinical features of NTM disease, regardless of treatment [3].

The severity of hemoptysis ranges from blood-tinged sputum to massive hemoptysis, and it can be life-threatening depending on the cause and severity [7]. In Korea, pulmonary tuberculosis is known to be the leading cause of hemoptysis, and there are many studies on pulmonary tuberculosis and hemoptysis [8, 9]. However, there are few data about hemoptysis among patients with NTM lung disease in Korea as well as in other countries. This study investigated the characteristics and prognosis of NTM lung disease with hemoptysis.

## Methods

### Study design and study subjects

This study was performed through a retrospective review of medical records at Ewha Womans University Mokdong Hospital, a tertiary referral hospital in Seoul, Korea. We reviewed 272 patients who had at least one NTM isolation in the sputum or bronchial lavage specimen culture between January 2006 and December 2016. Among them, 207 patients were diagnosed with NTM lung disease according to the ATS/IDSA diagnostic criteria [4]; 183 patients with available medical records were included in the final analysis. The enrolled patients were divided into two groups according to the occurrence of hemoptysis. Patients with hemoptysis received medical management when the amount of hemoptysis was low or vital signs were stable, and further management—such as bronchial artery embolization (BAE) and surgery—was considered if the hemoptysis persisted or the vital signs were unstable. We evaluated the development rate of hemoptysis, the characteristics of hemoptysis patients, and the complications that occurred among patients who underwent BAE.

### Data and definitions

Data for all enrolled patients were collected from the hospital’s electronic medical records. We recorded various demographic and comorbidity data, including sex, age, and body mass index, as well as the presence of diabetes mellitus (DM) and hypertension. We also noted the administration of antiplatelet and anticoagulation medication, which may be associated with hemoptysis. All enrolled patients underwent chest X-ray and chest computed tomography (CT) scans, which were reviewed retrospectively by the same chest radiologist (SS Shim). For the purposes of this study, we evaluated chest CT images from when hemoptysis was present for patients with hemoptysis; for patients without hemoptysis, we used the most recent follow-up chest CT images. Radiologic findings were classified as either nodular bronchiectatic type or fibrocavitary type. The extent of lung involvement was classified as either localized disease with involvement of 1 to 3 lobes or extensive disease with involvement of 4 to 5 lobes. Potential comorbidities were compared using the Charlson Comorbidity Index [10].

Hemoptysis was defined as the patient who underwent chest CT scan for hemoptysis evaluation. We excluded patients who did not undergo CT scan when defining hemoptysis. BAE was defined by actual embolizations; this definition did not include simple angiographies. The analysis included 33 consecutive patients who underwent BAE performed by two interventional radiologists (SY Choi, MW Kim). Medications, including antitussives, antibiotics, and tranexamic acid, were used for patients who had hemoptysis but did not undergo BAE.

### Statistical analysis

Results for all continuous variables are reported as mean ± standard deviation, and all categorical variables are reported as numbers and percentages. Between groups, continuous variables were analyzed using t-tests, and categorical variables were analyzed using the χ^2^ test and Fisher’s exact test. Multivariate analysis was performed using the results of initial univariate analysis and logistic regression. All statistical analyses were performed using SPSS version 23.0 (IBM Corp., Armonk, NY, USA). In all comparisons, a *P*-value of < 0.05 was considered statistically significant.

## Results

### Baseline characteristics

The baseline characteristics of enrolled patients from January 2006 to December 2016 are summarized in Table 1. Among 183 patients, there were 74 males (40.4%), and the mean age at diagnosis was 62.9 ± 12.7 years. *Mycobacterium intracellulare* (*n* = 64, 35%) was the most frequent cause of NTM infection, followed by *Mycobacterium avium* (*n* = 59, 32.2%), MABSC (*n* = 40, 21.9%). Fiberoptic bronchoscopy was conducted on 53% of patients for the diagnosis of NTM infection or evaluation of hemoptysis. Patients with the nodular bronchiectatic type of NTM disease were more common than those with the fibrocavitary type (*n* = 155, 84.7% vs. *n* = 28, 15.3%, respectively). Of the 155 patients with nodular bronchiectatic NTM disease, 113 had localized disease, and 42 had extensive disease. Of the 28 patients with fibrocavitary NTM disease, 12 had localized disease, and 16 had extensive disease. During a mean follow-up period of 48.7 ± 37.8 months, 78 patients (42.6%) developed hemoptysis, and 33 of 78 (42.3%) developed hemoptysis requiring BAE. No patients underwent surgery to treat hemoptysis.

**Table 1:** Demographic and clinical characteristics

Characteristics	*N* = 183
Male sex	74 (40.4)
Age at diagnosis, years	63.1 ± 12.7
BMI, kg/m^2^	20.0 ± 3
Diabetes mellitus	25 (13.7)
Hypertension	47 (25.7)
Antiplatelet or anti-coagulation	21 (11.5)
History of pulmonary tuberculosis	65 (35.5)
Hemoptysis	78 (42.6)
Occurrence of bronchial artery embolization	33 (18.0)
Surgical management of hemoptysis	0 (0)
Radiologic finding of NTM lung diseases	
Nodular bronchiectatic	155 (84.7)
≤ 3 lobes	113 (61.7)
> 3 lobes	42 (23.0)
Fibrocavitary	28 (15.3)
≤ 3 lobes	12 (6.6)
> 3 lobes	16 (8.7)
Distribution of chest CT	
≤ 3 lobes	125 (68.3)
> 3 lobes	58 (31.7)
Follow up duration, months	48.7 ± 37.8
NTM species identified from pulmonary specimens	
*M. avium*	59 (32.2)
*M. intracellulare*	64 (35.0)
*M. abscessus*	40 (21.9)
*M. kansasii*	6 (3.3)
*M. fortuitum*	2 (1.1)
*M. heckeshornense*	1(0.5)
*M. szulgai*	1(0.5)
*M. avium + M. intracellulare*	2 (1.1)
*M. avium + M abscessus*	4 (2.2)
*M. intracellulare + M. abscessus*	3 (1.6)
*M. abscessus + M. tuberculosis*	1 (0.5)

Results are presented as n (%) or mean ± standard deviation, unless otherwise indicated

*BMI* body mass index, *NTM* nontuberculous mycobacteria, *CT* computed tomography, *M. Mycobacterium*

### Comparison of groups

For comparison, enrolled patients were divided into two groups according to the occurrence hemoptysis (Table 2). There was no significant difference between the groups in terms of follow-up duration, the proportion of male sex, radiologic finding classification, extent of lung involvement, presence of pulmonary tuberculosis history, antiplatelet/anticoagulation therapy, and species of NTM. However, patients in the hemoptysis group tended to be younger than those without hemoptysis (mean age 65.7 ± 12.8 vs. 59.7 ± 11.8 years, *P* = 0.002). Additionally, patients without hemoptysis had a significantly higher DM prevalence than those with hemoptysis (18.1% vs. 7.7%, *P* = 0.043). In the multivariate analysis including sex, the presence of a pulmonary tuberculosis history, and variables with *P*-values < 0.1 from the univariate analysis, only age at diagnosis showed a statistically significant between-group difference (odds ratio: 0.969, 95% confidence interval: 0.944–0.996, *P* = 0.023).

**Table 2:** Comparison of groups with and without hemoptysis

	Univariate			Multivariate
	Without hemoptysis (*n* = 105)	With hemoptysis (*n* = 78)	*P*-value	*OR (95% CI)*
Male sex	47 (44.8)	27 (34.6)	0.167	1.211 (0.632–2.321)
Age, years, at diagnosis	65.7 ± 12.8	59.7 ± 11.8	0.002	0.969 (0.943–0.996)
BMI, kg/m^2^	20.0 ± 3.3	20.2 ± 2.7	0.758	
Diabetes mellitus	19 (18.1)	6 (7.7)	0.043	0.508 (0.179–1.441)
Hypertension	32 (30.5)	15 (19.2)	0.085	0.870 (0.398–1.903)
Follow-up duration, months	48.3 ± 36.1	49.2 ± 37.9	0.864	
Performed bronchoscopy	45 (42.9)	52 (66.7)	0.002	
Type of chest CT				
Nodular bronchiectatic	89 (84.8)	66 (84.6)	0.978	
Fibrocavitary	16 (15.2)	12 (15.4)		
Distribution of chest CT				
≤ 3 lobes	67 (64.4)	58 (74.4)	0.129	
> 3 lobes	38 (36.2)	20 (25.6)		
Antiplatelet/anticoagulation therapy	11(10.5)	10 (12.8)	0.646	
Liver disease	7 (6.7)	9 (11.5)	0.080	1.117 (0.668–1.869)
History of pulmonary tuberculosis	34 (32.4)	31 (39.7)	0.303	1.366 (0.726–2.572)
Charlson Comorbidity Index score	1.5 ± 1.8	1.3 ± 1.6	0.315	
NTM species identified				
*M. avium*	31 (29.5)	28 (35.9)	0.181	
*M. intracellulare*	44 (41.9)	20 (25.6)		
*M. abscessus*	20 (19.0)	20 (25.6)		
*M. kansasii*	4 (3.8)	2 (2.6)		
*M. fortuitum*	1 (1.0)	1(1.3)		
*M. heckeshornense*	0	1(1.3)		
*M. szulgai*	0	1(1.3)		
*M. avium + M. intracellulare*	0	2(2.6)		
*M. avium + M. abscessus*	3 (2.9)	1 (1.3)		
*M. intracellulare + M. abscessus*	1 (1)	2 (2.6)		
*M. abscessus + M. tuberculosis*	1(1)	0		

Results are presented as n (%) or mean ± standard deviation, unless otherwise indicated

*BMI* body mass index, *OR* odds ratio, *CI* confidence interval, *CT* computed tomography, *NTM* nontuberculous mycobacteria, *M. Mycobacterium*

### Analysis of group with hemoptysis

Of 78 patients with hemoptysis, 33 patients received BAE, and 45 patients received medical treatment (Table 3). All patients who received BAE showed immediate clinical improvement, and there were no patients who required surgery for hemoptysis. Among patients with hemoptysis, those requiring medical therapy and those requiring intervention therapy with BAE were not significantly different in terms of demographic characteristics, radiologic findings, extent of lung involvement, and comorbidity index. Additionally, there were no procedure-related complications or deaths, and there was no hemoptysis-related mortality. Eight of the 33 patients who underwent BAE subsequently underwent repeat BAE due to rebleeding, and the median interval between first and second BAE was 10 months (interquartile range 7.5–53.5 month). Four patients experienced recurrent hemoptysis within 12 months after the first BAE, and three of them required more than two more BAEs (total 8, 5, and 5 BAEs, respectively). On the other hand, 12 out of the 45 patients who received medical therapy experienced recurrence of their hemoptysis. Recurrent hemoptysis in both groups was mainly the nodular bronchiectatic type. However, patients in the BAE group were mainly infected with MABSC, and patients who received medical treatment were mainly infected with *Mycobacterium avium* complex. Table 4 shows the clinical characteristics among recurrent hemoptysis patients after BAE and medical therapy.

**Table 3:** Comparison of BAE and non-BAE subjects with hemoptysis

	Medical (*n* = 45)	BAE (*n* = 33)	*P*-value
Male sex	12 (26.7)	15 (45.5)	0.098
Age, years, at diagnosis	59.5 ± 11.7	60.0 ± 12.1	0.855
BMI, kg/m^2^	20.5 ± 2.6	19.8 ± 2.9	0.274
Diabetes mellitus	2 (4.4)	4 (12.1)	0.392
Hypertension	8 (17.8)	7 (21.2)	0.704
Performed bronchoscopy	28 (62.2)	24 (72.7)	0.466
Type of chest CT			
Nodular bronchiectatic	38 (84.4)	28 (84.8)	0.961
Fibrocavitary	7 (15.6)	5 (15.2)	
> 3 lobe distribution of chest CT	8 (17.8)	12 (36.4)	0.073
Antiplatelet/anticoagulation therapy	5 (11.1)	5 (15.2)	0.735
Liver disease	4 (8.9)	5 (15.2)	0.726
History of pulmonary tuberculosis	15 (33.3)	16 (48.5)	0.242
Charlson Comorbidity Index score	1.04 ± 1.1	1.6 ± 2.0	0.119
2 NTM pathogens identified	4 (8.9)	1 (3.0)	0.389
Complication after BAE		0	
Repeat BAE due to rebleeding		8 (24.2)	
Duration between first BAE and rebleeding^a^		10 (7.5–53.5)^a^	
Number of BAE trials			
1		25 (75.8)	
2		5 (15.2)	
5		2 (6.1)	
8		1 (3.0)	

Results are presented as n (%) or mean ± standard deviation, unless otherwise indicated

*BAE* bronchial artery embolization, *BMI* body mass index, *CT* computed tomography, *NTM* nontuberculous mycobacteria, *M. Mycobacterium*

^a^this value is presented as median (interquartile range) because of the small number of subjects

**Table 4:** Clinical characteristics of recurrent hemoptysis patients after BAE

Number	NTM pathogen	Interval to recurrence	Number of BAEs	Radiological type on chest CT	Distribution of chest CT	History of pulmonary TB	Antiplatelet/ Anticoagulation	CCI score
BAE group								
Case 1	*M. abscessus*	63 months	2	Fibrocavitary	> 3 lobes	Yes	No	1
Case 2	*M. abscessus*	8 months	8	Nodular bronchiectatic	> 3 lobes	No	No	2
Case 3	*M. intracellulare*	28 months	2	Nodular bronchiectatic	≤ 3 lobes	Yes	Yes	1
Case 4	*M. abscessus*	8 months	5	Nodular bronchiectatic	> 3 lobes	Yes	No	1
Case 5	*M. intracellulare*	3 months	5	Nodular bronchiectatic	≤ 3 lobes	Yes	No	1
Case 6	*M. avium*	62 months	2	Nodular bronchiectatic	≤ 3 lobes	No	No	0
Case 7	*M. abscessus*	7 months	2	Nodular bronchiectatic	≤ 3 lobes	No	Yes	2
Case 8	*M. abscessus*	12 months	2	Nodular bronchiectatic	> 3 lobes	No	No	2
Medical group								
Case 1	*M. abscessus*	47 months		Nodular bronchiectatic	≤ 3 lobes	No	No	2
Case 2	*M. abscessus*	70 months		Nodular bronchiectatic	≤ 3 lobes	No	No	1
Case 3	*M. avium*	9 months		Nodular bronchiectatic	> 3 lobes	No	No	3
Case 4	*M. avium*	2 months		Nodular bronchiectatic	≤ 3 lobes	No	No	0
Case 5	*M. avium*	88 months		Nodular bronchiectatic	≤ 3 lobes	No	No	0
Case 6	*M. avium*	66 months		Nodular bronchiectatic	≤ 3 lobes	No	No	2
Case 7	*M. avium*	133 months		Nodular bronchiectatic	≤ 3 lobes	Yes	No	0
Case 8	*M. avium*	75 months		Nodular bronchiectatic	≤ 3 lobes	Yes	No	1
Case 9	*M. intracellulare*	59 months		Nodular bronchiectatic	≤ 3 lobes	Yes	No	4
Case 10	*M. intracellulare*	4 months		Nodular bronchiectatic	≤ 3 lobes	No	Yes	2
Case 11	*M. abscessus*	19 months		Nodular bronchiectatic	≤ 3 lobes	No	No	3
Case 12	*M. avium*	2 months		Fibrocavitary	≤ 3 lobes	No	No	2

*BAE* bronchial artery embolization, *NTM* nontuberculous mycobacteria, *CT* computed tomography, *TB* tuberculosis, *CCI* Charlson Comorbidity Index, *M*. *Mycobacterium*

## Discussion

This study investigated the clinical characteristics, the development rate of hemoptysis requiring BAE, and the treatment of hemoptysis among NTM lung disease patients. Many patients with NTM lung disease experienced hemoptysis (42.6%) regardless of amount, and 18% of NTM lung disease patients required BAE. No significant risk factors for hemoptysis were determined other than relatively young age. Many patients (33/78, 42.3%) received nonmedical treatment for their hemoptysis, and BAE was preferred to surgical treatment. Furthermore, BAE was not associated with significant adverse events, and patients who experienced recurrence within 12 months were more likely to require BAE treatment.

Until now, NTM lung disease was known to cause hemoptysis through pulmonary parenchymal infection [11]; however, it is not considered a major cause of hemoptysis. Although the causes of hemoptysis vary from country to country—bronchitis, bronchogenic carcinoma, bronchiectasis, *Paragonimus westermani* infection, and pulmonary tuberculosis are known to be common causes [8, 12–14]. However, our findings demonstrated that many NTM lung disease patients experience hemoptysis requiring BAE.

Recently, the prevalence of NTM lung disease has been gradually increasing worldwide, and its onset is known to be associated with old age [1, 15, 16]. As the global population continues to age, and many countries already have aging or aged societies [17], the proportion of NTM lung disease is expected to increase, and the frequency of NTM lung disease-associated hemoptysis is expected to increase.

In the multivariate analysis, relatively young age was revealed as an independent risk factor. Unfortunately, this observation is difficult to explain. However, regarding pulmonary tuberculosis, some studies have reported that hemoptysis was significantly more common among younger people [18, 19]; the assumption is that the more aggressive immune response among younger patients may be more likely to induce hemoptysis [20]. Similarly, in our study, NTM lung disease may have also caused more hemoptysis among relatively young individuals via a more aggressive immune response. Additionally, our study showed that the occurrence and severity (requiring BAE or not) of hemoptysis among NTM lung disease patients was not associated with chest CT patterns, severity of involvement, or species of NTM pathogen. Although patients with cavities on chest CT were expected to experience severe hemoptysis as well as recurrence, there was no difference compared to those with bronchiectatic CT features. This may be because the great majority of the study sample did not have cavitary lung disease, or because bronchiectasis itself is a major risk factor for hemoptysis [9]. Therefore, clinicians need to consider the possibility of hemoptysis in young patients with NTM regardless of severity and extent of lung involvement.

BAE has been used as the first-line therapy, or a bridge to surgical treatment, for massive hemoptysis [14]. With improved technology, the overall success and immediate clinical success rates of BAE have been reported to be as high as over 90 and 73% to 99% [14]. Although several guidelines and studies have reported that NTM lung disease can cause hemoptysis, the treatment of hemoptysis has not been extensively detailed, with some studies briefly mentioning surgical treatment [3, 4, 21–23]. However, this study demonstrated that BAE is a good treatment option for hemoptysis in NTM lung disease, like other diseases which can cause hemoptysis [7]. There was immediate clinical success and no serious complications for all patients who underwent BAE during the period under review.

About one-quarter of BAE patients developed recurrent hemoptysis in this study. Some studies have reported recurrence in 10 to 20% patients over follow-up periods between 6 and 12 months [24–26]. Considering the duration (median 10 months) between first BAE and rebleeding in this study, BAE for hemoptysis in NTM lung disease showed a similar recurrence rate to BAE for hemoptysis in other diseases [24–26]. Causes of rebleeding include incomplete embolization, revascularization, or recanalization [24–26]. Some studies have found pulmonary tuberculosis and aspergillus to be risk factors for the recurrence of hemoptysis [27, 28]. However, although we did not perform the relevant statistical analysis due to the small sample size, the presence of lung damage from previous pulmonary tuberculosis did not appear to affect recurrence in this study. It was notable that most rebleeding patients were infected with MABSC and had nodular bronchiectatic radiologic findings on chest CT. Although this finding is hard to generalize due to the small number of patients with recurrent hemoptysis, considering that MABSC is relatively difficult to treat and can also progress with a fulminant course and that bronchiectasis features on chest CT include hypertrophy and tortuosity of the bronchial arteries [14, 29], these may be suggested as risk factors for the recurrence of hemoptysis.

This study had some limitations, including its retrospective design and that it was performed at a single center. Therefore, in our study, data regarding the rate of hemoptysis, hemoptysis volume, indication for BAE, and the techniques used for BAE—including late hemoptysis during the follow-up period after diagnosis—were not always available and may not be accurately reported. Additionally, we could not review the first presentations among the NTM lung disease patients or the role of bronchoscopy among these patients. This study did not reveal how NTM treatment affected the occurrence or recurrence of hemoptysis because we could not find consistent indications for NTM treatment in this study. Finally, subjects in this study had a higher prevalence of pulmonary tuberculosis history compared with other studies because Korea has a high prevalence of tuberculosis [1, 8, 30]. It is, therefore, difficult to generalize the results of this study to countries with low rates of tuberculosis. However, this study is meaningful because, to our knowledge, it is the first study of hemoptysis in NTM lung disease. As the prevalence of NTM disease increases, prospective studies about hemoptysis in NTM lung disease may be needed.

## Conclusion

NTM lung disease patients commonly experienced hemoptysis without specific risk factors except for relatively young age. Some patients with hemoptysis required BAE, the success rate of BAE was high, and there were no serious complications associated with BAE. Therefore, BAE could be considered a suitable treatment option for hemoptysis in NTM lung disease.

## Data Availability

The datasets generated and/or analyzed during the current study are not publicly available due to our IRB policy but are available from the corresponding author upon reasonable request.

## Abbreviations

ATS: American Thoracic Society
BAE: Bronchial artery embolization
CT: Computed tomography
DM: Diabetes mellitus
IDSA: Infectious Disease Society of America
MABSC: *Mycobacterium abscessus* complex
NTM: Nontuberculous mycobacteria
